# Exploring Pediatric Tele-Rheumatology Practices During COVID-19: A Survey of the PRCOIN Network

**DOI:** 10.3389/fped.2021.642460

**Published:** 2021-03-04

**Authors:** Y. Ingrid Goh, Danielle R. Bullock, Janalee Taylor, Rajdeep Pooni, Tzielan C. Lee, Sheetal S. Vora, Cagri Yildirim-Toruner, Esi M. Morgan, Nancy Pan, Julia G. Harris, Andrew Warmin, Kendra Wiegand, Jon M. Burnham, Fatima Barbar-Smiley

**Affiliations:** ^1^Division of Rheumatology, The Hospital for Sick Children, Toronto, ON, Canada; ^2^Child Health Evaluative Sciences, SickKids Research Institute, Toronto, ON, Canada; ^3^Division of Rheumatology, Department of Pediatrics, University of Minnesota, Minneapolis, MN, United States; ^4^Division of Rheumatology, Cincinnati Children's Hospital Medical Center, Cincinnati, OH, United States; ^5^Division of Allergy, Immunology and Rheumatology, Stanford Children's Health, Palo Alto, CA, United States; ^6^Division of Pediatric Rheumatology, Atrium Health Levine Children's Hospital, Charlotte, NC, United States; ^7^Department of Rheumatology, Baylor College of Medicine, Houston, TX, United States; ^8^Department of Rheumatology, Texas Children's Hospital, Houston, TX, United States; ^9^Division of Pediatric Rheumatology, Hospital for Special Surgery, Weill Medical College of Cornell University, New York, NY, United States; ^10^Division of Pediatric Rheumatology, Children's Mercy Kansas City, Kansas City, MO, United States; ^11^Cincinnati Children's Hospital Medical Center, Cincinnati, OH, United States; ^12^Division of Rheumatology, Children's Hospital of Philadelphia, Philadelphia, PA, United States; ^13^Division of Rheumatology, Nationwide Children's Hospital, Columbus, OH, United States

**Keywords:** telemedicine, pediatric rheumatology, telehealth, COVID-19, virtual platform, digital health (eHealth), health services research, virtual care

## Abstract

Healthcare providers were rapidly forced to modify the way they practiced medicine during the coronavirus disease 2019 (COVID-19) pandemic. Many providers transitioned from seeing their patients in person to virtually using telemedicine platforms with limited training and experience using this medium. In pediatric rheumatology, this was further complicated as musculoskeletal exams typically require hands-on assessment of patients. The objective of this study was to examine the adoption of telemedicine into pediatric rheumatology practices, to assess its benefits and challenges, and to gather opinions on its continued use. A survey was sent to the lead representatives of each Pediatric Rheumatology Care and Outcomes Improvement Network (PR-COIN) site to collect data about their center's experience with telemedicine during the COVID-19 pandemic. Quantitative data were analyzed using descriptive statistics, and qualitative data were thematically analyzed. Responses were received from the majority [19/21 (90%)] of PR-COIN sites. All respondents reported transitioning from in-person to primarily virtual patient visits during the COVID-19 pandemic. All centers reported seeing both new consultations and follow-up patients over telemedicine. Most centers reported using both audio and video conferencing systems to conduct their telemedicine visits. The majority of respondents [13/19 (68%)] indicated that at least 50% of their site's providers consistently used pediatric Gait Arms Legs and Spine (pGALS) to perform active joint count assessments over telemedicine. Over half of the centers [11/19 (58%)] reported collecting patient-reported outcomes (PROs), but the rate of reliably documenting clinical components varied. A few sites [7/19 (37%)] reported performing research-related activity during telemedicine visits. All centers thought that telemedicine visits were able to meet providers' needs and support their continued use when the pandemic ends. Benefits reported with telemedicine visits included convenience and continuity of care for families. Conversely, challenges included limited ability to perform physical exams and varying access to technology. Pediatric rheumatology providers were able to transition to conducting virtual visits during the COVID-19 pandemic. Healthcare providers recognize how telemedicine can enhance their practice, but challenges need to be overcome in order to ensure equitable, sustainable delivery of quality and patient-centered care.

## Introduction

The coronavirus disease 2019 (COVID-19) pandemic triggered an international call for physical distancing, which limited patients' access to healthcare. As a result, healthcare providers explored telemedicine as an alternative or complementary method of delivering medical care (1, 2). The pediatric population, specifically those with chronic diseases, have higher medical needs, necessitating frequent visits to their medical provider (3). Due to the nature of their underlying diseases and treatment with immunosuppressive medications, children with rheumatic conditions require ongoing medical care for both physical exam assessment and laboratory studies (3).

Limited access to pediatric rheumatology care is an established issue, which is further exacerbated by the shortage of providers (4). Although there was little infrastructure to support virtual visits, telemedicine was proposed as a solution to improving access to pediatric rheumatologists prior to the COVID-19 pandemic (5–7). While previous telemedicine studies in other areas of medicine including adult rheumatology have shown promise, there are limited published reports regarding its use in the pediatric rheumatology setting (8–12). Piga et al.'s (13) systematic review on feasibility, effectiveness, and patient satisfaction with telemedicine for patients with rheumatic disease identified three studies involving patients with juvenile idiopathic arthritis (JIA). Two of the studies were self-management studies and one was an education study; therefore, remote disease activity assessment was not performed on these studies (14–16). An abstract published in 2014 surveying 77 pediatric rheumatology practices reported that seven sites had telemedicine capabilities, but only three sites actively used telemedicine to see patients (17). Another study at one center reported that families preferred in-person to telemedicine visits, though most of the respondents were unfamiliar with telemedicine (9). The rate of telemedicine acceptance appeared to increase with greater familiarity with this medium (9). Despite the evidence suggesting telemedicine could result in potential cost savings, the adoption of telemedicine remained low for the reasons mentioned above (8). An abstract published in 2018 reported on the experience of providing pediatric rheumatology care over telemedicine using a mixed model (10). Patients traveled to a site close to their home with telemedicine capabilities, where they connected virtually with their pediatric rheumatologist while having a hands-on joint disease activity assessment performed by their local Advanced Clinician Practitioner in Arthritis Care (ACPAC) practitioner (10, 18). Decreased cost and burden associated with travel, as well as increased access to care and patient satisfaction, were noted (10).

The COVID-19 pandemic forced healthcare providers, including pediatric rheumatology providers, to rapidly shift to virtual care. Many of the previous barriers to telemedicine, such as reimbursement and regulatory concerns, were abruptly lifted to allow for its accelerated adoption (19). The practice of telemedicine facilitated uninterrupted medical care while abiding by physical distancing requirements. The forced, expedited adoption of telemedicine across pediatric rheumatology clinics came with its challenges. The recognition of these challenges and barriers prompts the identification of potential solutions that will improve future delivery of care over telemedicine. The objective of this study was to examine the adoption of telemedicine into pediatric rheumatology practices during the COVID-19 pandemic, to assess its benefits and challenges, and to gather opinions on its continued use.

## Materials and Methods

The Pediatric Rheumatology Care and Outcomes Improvement Network (PR-COIN) is a quality improvement collaborative learning network of 21 pediatric rheumatology medical centers and parent/patient stakeholders across the United States and Canada (20). Together, they partner to identify and close gaps in healthcare for children with rheumatic diseases by leveraging quality improvement science and to bring research discoveries to patient care promptly (20). In addition, the Network strives to disseminate the knowledge gained to the wider community through education and publication of results (21). Participating sites are focused on improving the outcomes of care for children with rheumatic diseases (21).

In light of the rapid adoption of telemedicine in pediatric rheumatology, members of the PR-COIN Tele-Rheumatology Workgroup conducted an electronic survey. The main goal of this survey was to gather information reflecting each center's experiences during the COVID-19 pandemic, specifically related to telemedicine, including their rates of adoption, how visits were being conducted, and their opinions of seeing patients using this medium (see Supplementary Material for survey).

The survey was sent to the lead investigator at each PR-COIN center. They were requested to complete the survey within a 1 week period during June 2020. Survey data were collected and managed using REDCap® electronic data capture tool (22, 23). REDCap® is a secure, web-based software platform designed to support data capture for research studies, providing (1) an intuitive interface for validated data capture, (2) audit trails for tracking data manipulation and export procedures, (3) automated export procedures for seamless data downloads to common statistical packages, and (4) procedures for data integration and interoperability with external sources (22, 23).

Quantitative data were analyzed using descriptive statistics. Two independent reviewers analyzed the qualitative data using grounded theory to provide understanding of concepts and ideas emerging from the survey.

PR-COIN collaborative activities are covered under an umbrella Institutional Review Board (IRB) protocol, including member surveys that are used as part of continuing quality improvement.

## Results

### Center Demographics

The survey was completed by 19 of 21 (90%) PR-COIN centers, but only 18 of the surveys had complete responses. Centers varied in the size and composition of their team (range: 4–33 members at each site) (Table 1). Teams included pediatric rheumatologists, trainees, nurses, practitioners (nurse/ACPAC), allied health professionals (e.g., medical assistant, social workers, dietitian, physical therapist, occupational therapist), and physicians from other subspecialties such as adolescent medicine and dermatology. The smallest site was composed of four pediatric rheumatologists, whereas the largest site was composed of 11 pediatric rheumatologists, one nurse practitioner, two medical assistants, six nurses, six fellows, four physical therapists, two occupational therapists, and one social worker.

**Table 1:** Composition of Healthcare Team at PR-COIN Sites.

**Type of Members on Healthcare Team**	**Number of Sites**
Pediatric rheumatologists, trainees, nurses, practitioners (nurse/ACPAC)	3
Pediatric rheumatologists	2
Pediatric rheumatologists, trainees, nurses, allied health (medical assistants)	2
Pediatric rheumatologists, nurses, practitioners (nurse/ACPAC), allied health (medical assistants)	2
Pediatric rheumatologists, nurses, allied health (physical therapist/practical nurse/social worker)	2
Pediatric rheumatologists, trainees, nurses	2
Pediatric rheumatologists, nurses	1
Pediatric rheumatologists, trainees	1
Pediatric rheumatologists, nurses, allied health (social worker, physical therapist), specialists from other departments	1
Pediatric rheumatologists, trainees, practitioners (nurse/ACPAC), allied health (medical assistant)	1
Pediatric rheumatologists, trainees, nurses, practitioners (nurse/ACPAC), allied health (medical assistant/physical therapist/occupational therapist/social worker)	1
Pediatric rheumatologists, trainees, practitioners (nurse/ACPAC), nurses, allied health (physical therapist/social worker/dietitian), specialists from other departments	1

*ACPAC, Advanced Clinician Practitioner in Arthritis Care; PR-COIN, Pediatric Rheumatology Care and Outcomes Improvement Network*.

### Telemedicine Adoption and Utilization

**Figure 1:**
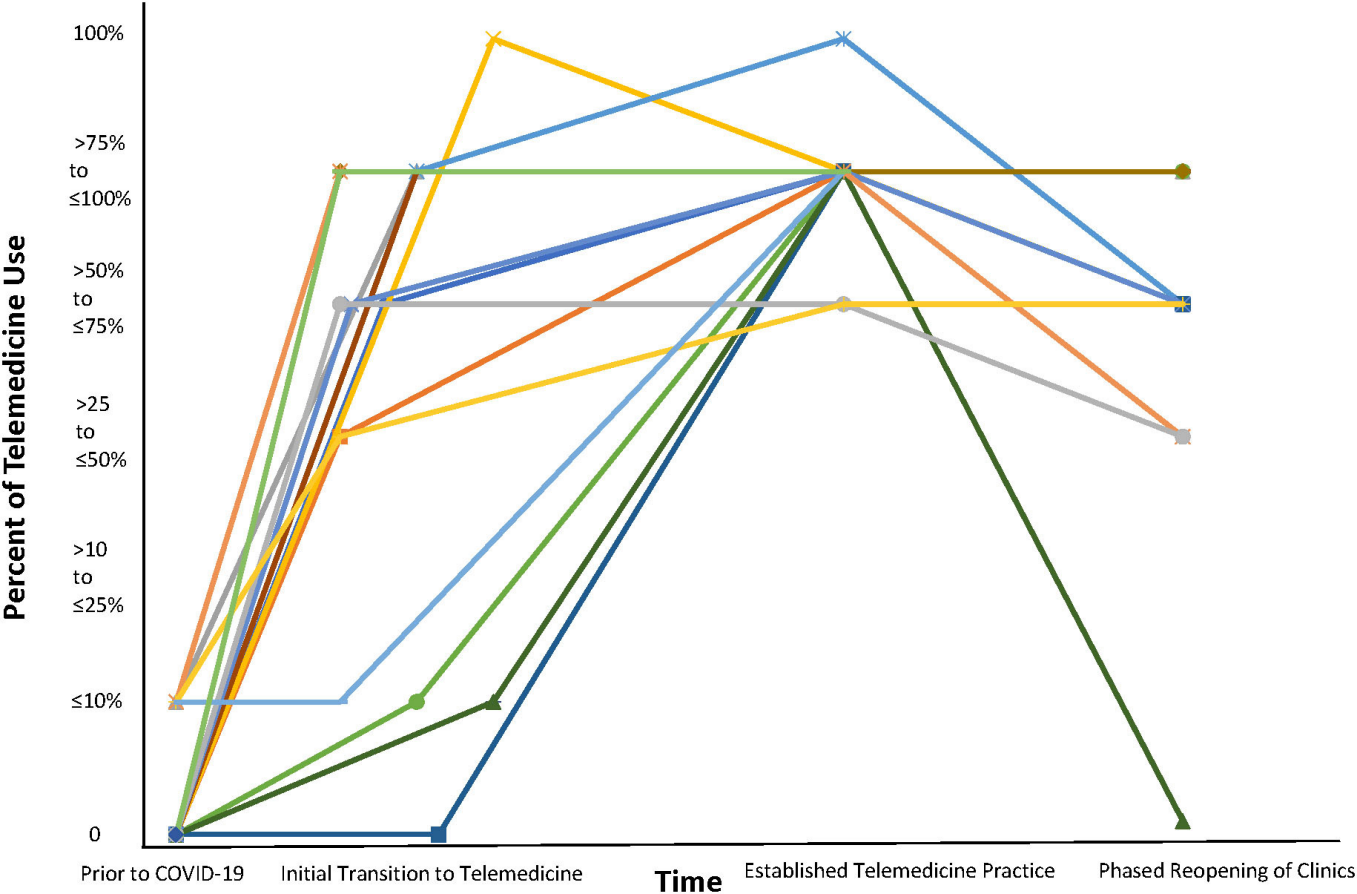
Use of Telemedicine Prior to and During the COVID-19 Pandemic. Each color represents a different center.

Of the 18 centers with completed responses, only four centers (21%) reported conducting telemedicine visits prior to the COVID-19 pandemic. However, telemedicine was previously utilized to service <10% of the four center's population. All 18 responding sites indicated that they were able to successfully adopt telemedicine visits during the COVID-19 pandemic. The switch from in-person to telemedicine services occurred mid-March for 10/18 (55%) of the centers, while the remaining centers switched mid to late March (Figure 1). At its peak, 16/18 (89%) reported seeing 75–100% of visits were conducted using telemedicine. As some sites began phased reopening (seeing patients in person) during late spring of 2020, the use of telemedicine subsequently decreased. All sites reported using telemedicine to see both new referrals and follow-up patients.

### Telemedicine Platforms

The most commonly reported platform used to conduct telemedicine visits was Zoom (7/19), followed by American Well (5/19). Sites also indicated that they used Microsoft Teams, FaceTime, Doximity, and Ontario Telemedicine Network (OTN) (2/19). Other rarely reported platforms included Jabber, WhatsApp, SBR Health, Bluejeans, and WebEx (1/19). The majority of sites (11/19) reported using MyChart as their patient portal. This was followed by HealthELife (3/19), FollowMyHealth (2/19), and one unspecified patient portal.

### Type of Telemedicine Visits

**Figure 2:**
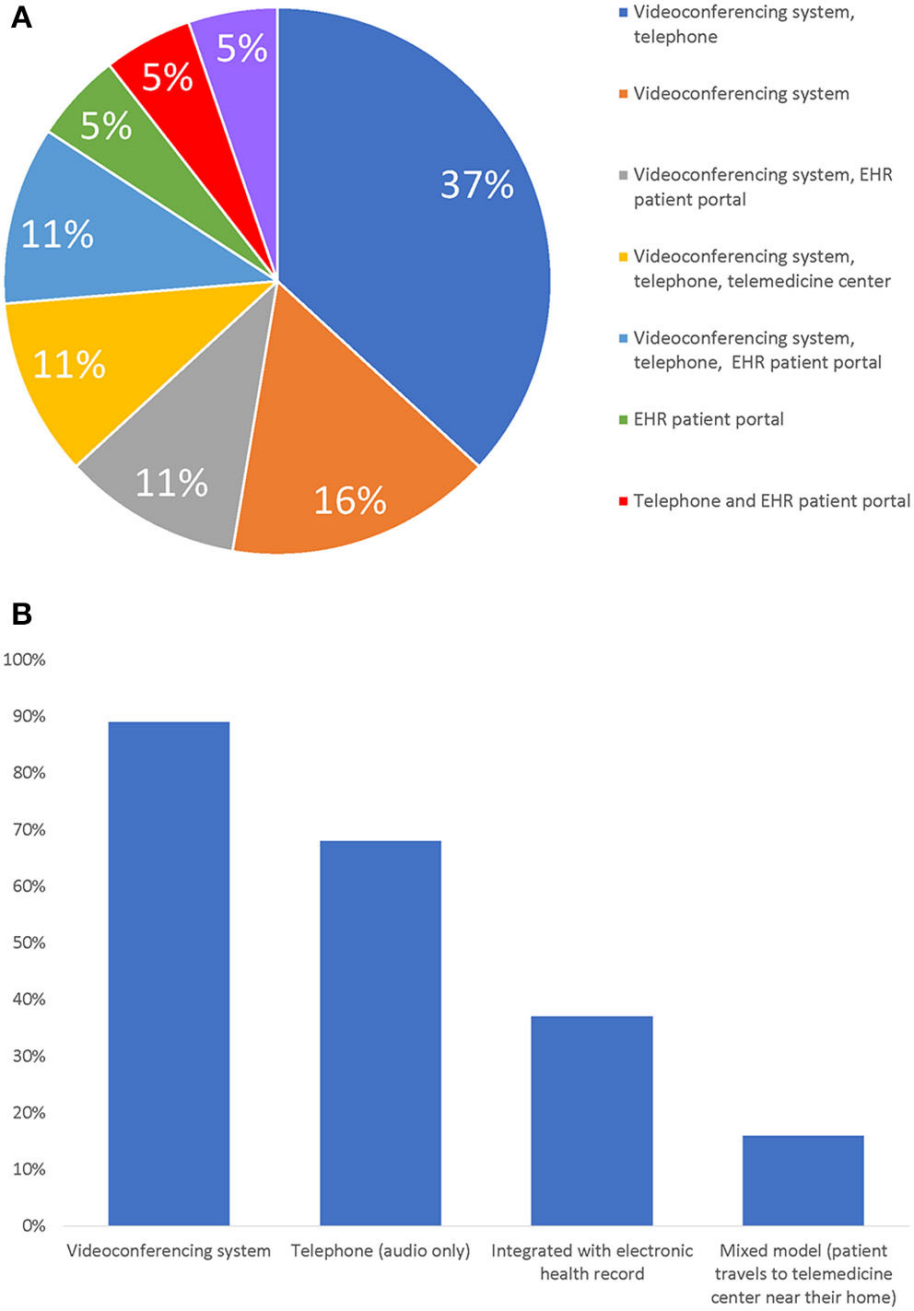
Mediums Used to Perform Telemedicine Visits. **(A)** Medium Usage by Sites. **(B)** Sites Usage of Mediums. Note: Bar graph answers are not mutually exclusive.

All sites reported their ability to conduct virtual visits with both audio and visual features (Figure 2). Here, 12/19 (63%) sites reported using videoconferencing systems, 5/19 (26%) reported using a combination of both videoconferencing systems and electronic health record (EHR) patient portals, while 2/19 (11%) sites reported using only their EHR patient portal. Moreover, 13/19 (68%) sites reported also conducting audio-only visits. Also, 3/19 (16%) sites reported using mixed models where patients traveled to a site close to their home with audio and video telemedicine capabilities. These three sites were using this mixed model prior to the COVID-19 pandemic.

### In-Person vs. Telemedicine Visit

When providers were asked about reasons to see a patient in person vs. using telemedicine, respondents indicated a patient having active or worsening disease and requiring additional medical care such as hospitalization or joint injections, coordinating collaborative care, or upon patient request (Table 2).

**Table 2:** Reasons for In-Person Visit Preferred Over Telemedicine Visit.

Joint injection	89%
Anticipate hospitalization	74%
Worsening condition	68%
Evidence of new rheumatic disease	63%
Active disease	58%
Parent request/desire	53%
New patient	47%
Other	16%
Need for laboratory visits	5%

### Effect on Patient Volume

The majority of centers [14/19 (74%)] reported that their patient volumes had decreased as a result of switching to telemedicine during COVID-19, while 4/19 (21%) reported that their volumes were about the same. One site was not certain about whether their patient volumes had been affected.

### Assessment of Joint Disease Activity

**Figure 3:**
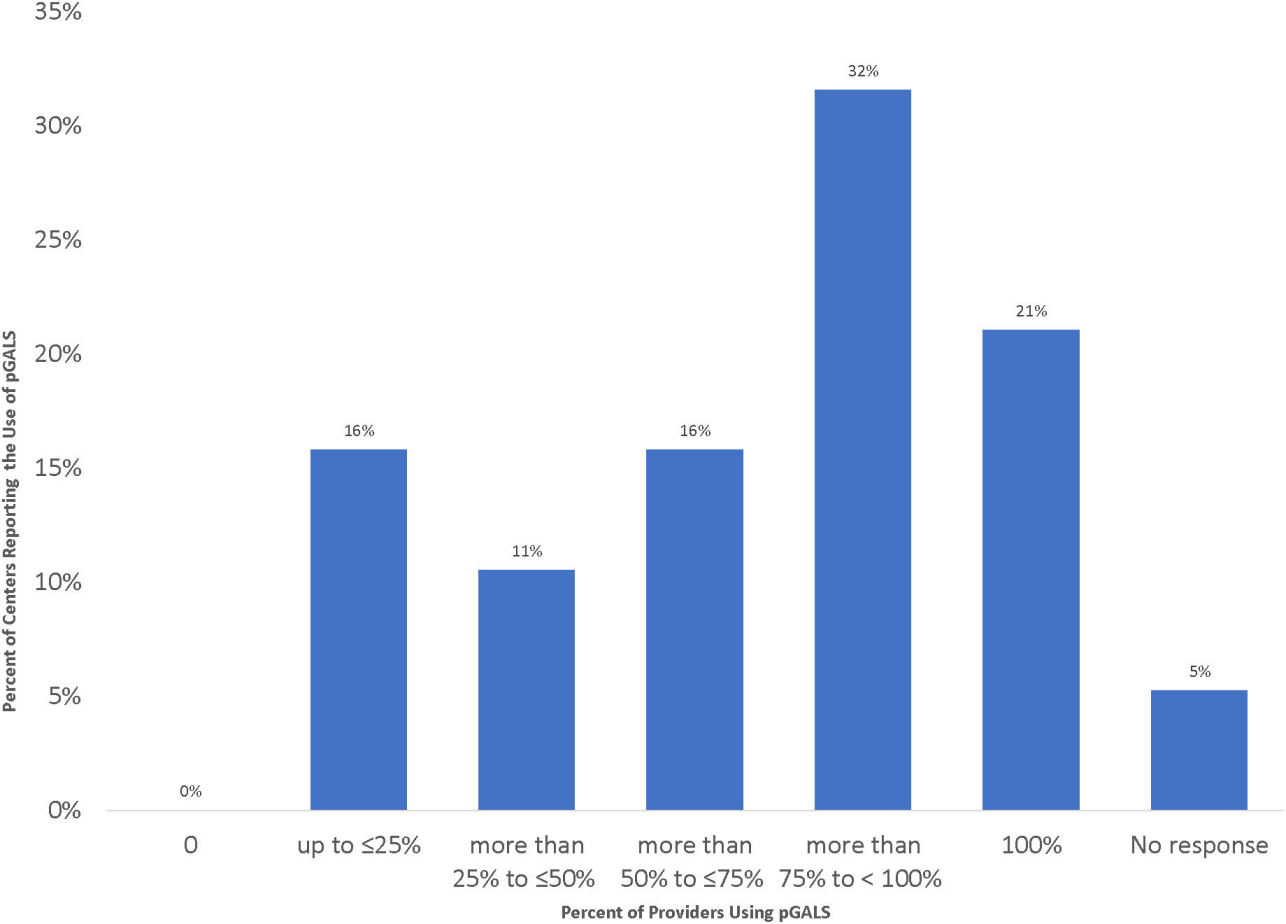
Percentage of Providers from Each Site Using pGALS to Assess Joint Disease Activity during Telemedicine Visits.

Most centers [13/19 (68%)] reported that at least 50% of their providers consistently used the pediatric Gait, Arms, Legs, Spine tool (pGALS) to perform joint activity assessments in patients with JIA (Figure 3). The pGALS is a structured musculoskeletal exam that has been used and validated in multiple languages to identify musculoskeletal abnormalities in children during in-person assessments (24).

### Patient-Reported Outcomes

Patient-reported outcomes (PROs) were collected by most sites (Table 3). Here, 15/19 (79%) sites reported collecting duration of morning stiffness. Multi-item PROs questionnaires, e.g., Child Health Assessment Questionnaire (CHAQ), or Patient-Reported Outcomes Measurement Information System (PROMIS) measures were collected by a small minority of centers (5–11%). The majority of sites reported verbally collecting PROs during the telemedicine appointment [16/19 (84%)]. In addition, 3/19 (16%) sites reported collecting PROs using their patient portals, 2/19 (11%) sites reported collecting PROs using e-mail, and 1/19 (5%) sites indicated they also had a custom system built during the COVID-19 pandemic to collect PROs. The PRO completion rate varied widely, with 5/19 (26%) sites reporting <50% completion, 5/19 (26%) sites reporting 50–75% completion, 5/19 (25%) sites reporting 76–100% completion, and the four remaining sites did not respond to this question. PROs that were able to be obtained verbally, such as morning stiffness or patient global assessment, were more reliably collected than Patient-Reported Outcome Measures (PROMs), which are generally longer validated questionnaires.

**Table 3:** Patient-Reported Outcomes Documented During Telemedicine Visit.

Morning stiffness	79%
Patient global assessment	68%
Pain intensity	58%
Patient self-reported joint count	26%
Do not collect any patient-reported outcomes (PROs)	11%
Child Health Assessment Questionnaire (CHAQ)	11%
Other	11%
Patient-Reported Outcomes Measurement Information System (PROMIS)	5%
Juvenile Arthritis Multidimensional Assessment Report (JAMAR)	5%
Pediatric Quality of Life Inventory™ (PedsQL)	0%

### Documentation

The reliability of providers documenting items is described in Table 4. Medication reconciliation, medication refills, and date of last eye examination were the items that were the most frequently documented. Conversely, height and weight were the least documented items.

**Table 4:** Items Documented During a Telemedicine Visit.

Medication reconciliation	95%
Medication refills	95%
Date of last eye examination	95%
Visit conducted using telemedicine	79%
Allergy review	79%
Laboratory results	79%
Physician Global Assessment	74%
Patient Global Assessment	74%
Joint count	68%
Patient-Reported Outcomes	53%
Disease activity (e.g., JADAS)	42%
Treatment target(s)	37%
Weight	26%
Height	0%

### Items Patients Received Prior to the Telemedicine Visit

The instructions provided to patients to prepare for their telemedicine visit varied from site to site. Consent was also obtained by some sites prior to their appointment. Patient instructions were e-mailed, mailed, or provided verbally. Patients would receive an e-mail link for the telemedicine appointment or notification by their patient portal. Two sites offered mock visits to ensure that patients knew how to connect. Some nurses and medical assistants are connected with patients prior to their visit to gather pre-visit information.

### Items Patient Received After the Telemedicine Visit

The majority of respondents indicated that patients received prescriptions 18/19 (95%), referrals 16/19 (84%), and after-visit summary 13/19 (68%) at the end of their telemedicine appointment. Additional items that were provided included requisitions for external labs [4/19 (21%)], physiotherapy resources [2/19 (11%)], requisition for external diagnostic imaging [1/19 (5%)], and disease-specific information [1/19 (5%)].

Patients generally received these materials through their patient portal [12/19 (63%)] or by mail [12/19 (63%)]. Also, 8/19 (42%) sites reported sending this information by e-mail, and 3/19 (16%) indicated that they provided it by fax. One respondent indicated that pertinent items were faxed directly to the recipient, e.g., pharmacy or other clinics.

### Research During Telemedicine Visits

With respect to conduct of research activities over telemedicine, 7/19 (36%) sites reported conducting research activity during this period, while 8/19 (42%) reported that they were unable to conduct research. Of the sites reporting the ability to conduct research during the COVID-19 pandemic, only 2/7 (14%) sites reported being able to obtain consent over telemedicine. Most of the research that continued was follow-up visits for registries where information could be abstracted from charts or clinical personnel were able to assist with a portion of the research process. Coordinating research during this time relied more heavily on communication with the clinical team and the clinical team's willingness to assist with activities.

### Benefits and Challenges to Use of Telemedicine

Benefits noted with telemedicine included improved convenience, no need to travel, continuity of care for families who were hesitant to have in-person appointments, and the ability to see patients in their natural environment (Table 5). Challenges noted with telemedicine included limited ability to perform physical exams, difficulties assessing disease activity, and difficulties accessing and utilizing technology (Table 6).

**Table 5:** Benefit of Telemedicine Visits.

No travel	95%
More convenient	84%
Continuity of care for families who are hesitant to come for in-person visits	84%
Less cancellations/no shows	58%
Decreased patient wait times (i.e., shorter time to schedule an appointment)	53%
Decreased clinic visit length (i.e., time spent with healthcare provider)	42%
Other (e.g., opportunity to observe a patient's home environment)	5%

**Table 6:** Challenges of Telemedicine Visits.

Limited ability to perform physical exams	89%
Assessing disease activity	84%
Access to technology	68%
Safety labs being performed at recommended interval	53%
Adequate Internet bandwidth	47%
Providing multidisciplinary care	42%
Patient education	32%
Communicating after-visit instructions/making follow-up visits	26%
Licensure	11%
Reimbursement	5%
Other-Miscellaneous technological issues for the patients/providers	5%

All respondents agreed telemedicine visits met both provider and patient needs. All respondents also indicated that they believed that the use of telemedicine visits should continue following the resolution of the COVID-19 state of emergency. At the time of survey, most centers 14/19 (74%) felt that <50% of established patients and 15/19 (79%) new patients could be safely and effectively seen over telemedicine moving forward.

## Discussion

Telemedicine has facilitated the continuity of care to pediatric rheumatology patients while reducing the risk of transmission of COVID-19 among healthcare providers, patients, and caregivers. To our knowledge, this is the first survey assessing the change in telemedicine practices in pediatric rheumatology due to the COVID-19 pandemic. Not only has telemedicine facilitated the continuity of care in pediatric rheumatology, but it has also been successfully adapted by other pediatric subspecialties including adolescent medicine, otolaryngology, and sleep medicine (25–27).

In light of the need to quickly respond during the pandemic, a variety of platforms were used to conduct telemedicine visits. With the adaptation and normalization of telemedicine into clinical practice, healthcare teams have since moved toward ensuring that telemedicine visits are conducted using private and secure [e.g., Health Insurance Portability and Accountability Act (HIPAA)/Personal Health Information Protection Act (PHIPA) compliant] healthcare information exchange platforms. Some institutions have invested in infrastructure to conduct these visits such as webcams and software that integrate virtual visits into patients' EHRs. Although the enactment of the National Emergencies Act in the United States and telehealth expansion in Canada helped overcome some barriers of reimbursement and HIPAA, it did not address the issue of reimbursement and providing telemedicine services to patients residing in another state (interstate licensure) or another country (28, 29).

Although our results indicated that all sites had the ability to conduct visits with audio and video capability, we do not know what proportion of visits were conducted audio-only consultations vs. audio and video consults.

Despite the availability of telemedicine, providers recognized circumstances where patients should be seen in person, suggesting that there may be a need to systematically triage patients to determine whether they should be seen in person or over telemedicine. This process may require additional time and preparation of the healthcare and administrative team. To our knowledge, there currently are no recommendations or guidelines on how to best triage pediatric rheumatology patients, which may warrant future investigations.

The reasons for changes in patient volumes during the COVID-19 pandemic are not specifically known. Possible explanations for decreased volumes include family reluctance to leave home during the pandemic, declined visit bookings, limited access or resources of families to conduct telemedicine visits, and caregiver's occupation and changes in work schedule. It is also not clear whether telemedicine visits are longer in duration or if the learning curve of conducting telemedicine visits affected scheduling volumes. Furthermore, patients may have limited ability to be seen over telemedicine because they might not have the bandwidth, resources, or knowledge of how to access telemedicine visits. We do not know how many new referrals each site receives annually or how many patients with JIA are serviced annually, therefore we cannot predict how large of a clinical volume is seen by healthcare providers at each site, which may affect their comfort level with using telemedicine.

Although most centers reported that some of their providers used the pGALS to assess joint activity, this tool has not been validated for use over telemedicine. As a result, healthcare providers may not be confident in the accuracy of active joint activity assessment when utilizing this tool over this telemedicine. The instrument also may not be able to identify small effusions or detect active joints in young children when used over telemedicine. Recently, Shenoi et al. (30) proposed the Video-pGALS (an adapted version of the pGALS), but this has not been validated and was created using input from a small group of pediatric rheumatologists. Additional research needs to be undertaken to determine whether this tool can accurately assess joint disease activity in a virtual setting. If it is determined that this tool is valid, it could enable the standardization of care over telemedicine. Conversely, if it is not valid, pediatric healthcare providers will need to seek other means of performing accurate joint disease activity assessments.

Given that our survey found that about half of the sites' teams used pGALS to assess their patients over telemedicine, it is possible that not all healthcare providers are aware of or trained in the use of the pGALS joint activity assessment tool. As such, there is an opportunity to train providers so they are aware of tools that may assist them during their physical assessment of patients. Similarly, should a tool be validated for performing assessment over telemedicine, it is important that the knowledge be disseminated to educate providers, which will, in turn, standardize care among pediatric rheumatology patients over telemedicine.

PROs are an integral part of routine clinical practice and contribute to a myriad of studies (31). The documentation of patient global assessments was reportedly low. This may be due to several factors including the reliance of the healthcare provider to ask the patients to rank the state of their rheumatic condition on a scale of 0–10 and then documenting the response. During the initial adoption of telemedicine, healthcare providers may have prioritized learning how to provide care using this medium and, therefore, may have elected to focus on completing items that they deemed to be a higher priority. With increased familiarity of delivering care over telemedicine platforms, it is possible that PRO collection and documentation have increased. Furthermore, as a proportion of patients will continue being seen over telemedicine after the pandemic is over, it would be worthwhile to implement a method to reliably collect PROMs for these visits.

The variability in the documentation of items during telemedicine visits compared to in-person visits may be due to several factors. Some variations may be accounted for by local billing, compliance, and/or institutional requirements. It appeared that information that the providers had more ease and control of obtaining, such as medication reconciliation, was more reliably documented, whereas other pieces of information that required some effort of patients and their caregivers (e.g., measuring height) or other measuring tools (e.g., weight scale) were less reliably documented. As telemedicine will continue to serve a proportion of patients, healthcare providers should consider how to equip families that may not have a scale or, less commonly, a stadiometer, on how to reliably collect these measurements. Providing educational resources to patients and families as well as communicating alternative methods to accomplish these measurements might be a feasible option. Most families have access to smartphones where they could download applications that may help them perform measurements or access websites, such as the Centers for Disease Control and Prevention (CDC), which have instructional guidelines (32, 33). Clinical support tools (such as Smart Phrases, note templates, and billing templates) embedded in the EHR may assist in reminding healthcare providers to document exam elements during telemedicine visits.

Given that everyone was expected to adapt to telemedicine rapidly and the lack of guidance documents at the outset of the pandemic, instructions provided to patients (if any were provided) varied in everyone's practice (even within sites). Since then, some guidance documents have been developed, but there are none specific to pediatric rheumatology (34). It would be worthwhile to look at the current practices among different sites to collectively learn from each other and create a best practice document. Surprisingly, some caregivers do not realize that patients needed to be present for telemedicine appointments or understand the importance of creating an environment to conduct the visit. The development of a best practice document with unified expectations and instructions may assist patients and caregivers to better prepare for their appointment.

Providing requisitions for external laboratory and diagnostic testing after the telemedicine visit offers patients more convenience as they can maintain continuity of care while not traveling too far from their home. Only one site reported providing patients with disease-specific information. It is not clear whether this was because it was an open-ended question and respondents did not think of this answer, or whether other providers did not have digitized resources to send to their patients. With the shift to telemedicine, healthcare teams should consider amassing digital resources that they can provide to their patients and families.

Many institutions limited research activities during the COVID-19 pandemic to reduce the number of staff entering their facilities as well as prioritize the ethical approval of COVID-19-related research activities (35). To ensure that scholarly work continues, it is important to establish mechanisms in which research can be performed over telemedicine (36). This will require developing and operationalizing a plan that is amenable to both healthcare and research teams. In addition, using virtual workflows such as digital consent forms and electronic case report forms will facilitate research activities over telemedicine. Naturally, there will be additional costs associated with these changes, and funders of research will need to avail additional funding to facilitate this transition (35). Researchers will need to include a telemedicine/virtual visit component within their future research budgets. Furthermore, to ensure diversity and inclusiveness, researchers will also need to consider ways to facilitate the participation of individuals who do not have access to technological tools.

Given that patients did not have to travel to attend their telemedicine visit, households saved time and money associated with traveling to see their healthcare provider. Not much is about the cost of pediatric rheumatology telemedicine visits, e.g., Internet data compared to in-person visits, e.g., traveling. It would be worthwhile to explore the economic impacts of these visits. Telemedicine visits offer an alternative solution to overcoming clinician shortages, especially in rural and other underserved populations. In pediatric rheumatology, where there is a clear workforce shortage and states without providers, there is a distinct opportunity to reduce travel-associated costs (8, 37) and ensure regularity of follow-up for patients, which is critical in pediatric chronic care. The convenience of telemedicine could have potentially brought back patients who were not seen in a long time because they were in remission and did not want to lose time and money to travel to their healthcare provider only to be told they were fine. As such, the use of telemedicine can improve the outcomes of patients with pediatric rheumatic conditions, as it increases access to, as well as facilitates, continuity of care.

The challenge of telemedicine is that it may be not be equally accessible to everyone. There remain certain regions with poor bandwidth and minimal Internet services (38). Due to socioeconomic circumstances, some families may not be able to afford the cost associated with these types of appointments. Further work is needed to elucidate the effect of socioeconomic status, language, broadband availability, and technologic literacy on patient access.

Although all respondents indicated that telemedicine met their needs, future work should consider assessing their satisfaction with telemedicine visits. Another item worth assessing is the learning curve associated with telemedicine visits or any additional stress associated with the switch to this medium.

The limitations of our study include that the participants were North American pediatric rheumatologists who were members of PR-COIN, therefore limiting the generalizability of our findings. PR-COIN sites are generally large pediatric research centers that have extensive experience conducting collaborative research. Future studies could be conducted on an international level to assist with the generalizability of the results. In addition, since this was distributed to PR-COIN site leaders, we are uncertain whether the results accurately reflect the opinions of all the providers at their site or whether they were responding based on personal experience. For sites reporting the use of telemedicine prior to the COVID-19 pandemic, we do not know whether this prior experience was equal among all staff members and whether this facilitated a quicker transition to telemedicine for the remainder of their practice. Furthermore, as this survey asked providers to reflect at a single point in time, opinions may have shifted over time with increased experience with the use of telemedicine. We are currently developing a follow-up survey to see whether healthcare providers' opinions have changed with time and experience and identify what novel ideas and tools have emerged ~1 year after mass the adoption of telemedicine. This will enable us to identify best practices. With the identification and implementation of best practices, future studies will be able to observe how disease monitoring over telemedicine changes over time.

Our survey only asked about the use of pGALS as an assessment tool and did not inquire about other tools providers used to assess joint disease activity over telemedicine. We plan to identify other tools that healthcare providers are using to assess joint disease activity, so that if a reliable method is uncovered, the knowledge can be shared with pediatric rheumatology healthcare providers.

Finally, this survey was only administered to healthcare providers and not patients and caregivers. Therefore, we do not have an understanding of telemedicine visits from their perspective. Future projects should include patients' and caregivers' perspectives in order to help understand their needs and barriers and what influences their decisions when selecting appointment mediums. It would also be informative to know how comfortable caregivers would feel if they were asked to actively assist in the physical examination process.

## Conclusions

The COVID-19 pandemic accelerated the change to delivering care over telemedicine. This survey indicates that rapid adaptations occurred to facilitate the implementation of pediatric rheumatology clinical care and research over telemedicine in response to the COVID-19 pandemic. In doing so, they were able to continue providing medical care to pediatric rheumatology patients despite the physical distancing requirements. Given that all sites were able to transition to providing care over telemedicine, the prospect of continuing this practice in the future is highly likely given that most institutions have added resources and infrastructure during the COVID-19 pandemic. We identified specific challenges healthcare providers faced when conducting visits over telemedicine, such as the limited number of available tools to reliably perform assessments, the lack of certainty with these evaluations, and ensuring that patients had access to technology in order to conduct telemedicine visits. Further research is needed to identify and validate tools that can reliably be used to perform assessments over telemedicine, identify mechanisms to improve provider documentation, identify ways to improve the collection of PROs, create standardized instructions to better prepare patients for a successful telemedicine visit, and identify ways to best integrate research visits along with telemedicine visits. Most importantly, we need to ensure that telemedicine can be delivered in a safe, supportive, and accessible way to pediatric rheumatology patients and their families.

## Data Availability Statement

The raw data supporting the conclusions of this article will be made available by the authors upon request.

## Ethics Statement

PR-COIN collaborative activities are covered under an umbrella Institutional Review Board (IRB) protocol at Cincinnati Children's Hospital Medical Center. Member surveys are used as part of continuing quality improvement. Written informed consent to participate was therefore not required for this study in accordance with the institutional requirements.

## Author Contributions

YG, NP, FB-S, JH, SV, AW, JB, JT, CY-T, and EM substantially contributed to the conception or design of the work, acquisition, analysis, or interpretation of data for the work. YG, FB-S, DB, JT, RP, and TL drafted the work. YG, FB-S, JT, SV, CY-T, EM, DB, NP, JH, TL, RP, JB, AW, and KW revised the work critically for important intellectual content. All authors approved the final version to be published and agreed to be accountable for all aspects of the work in ensuring that questions related to the accuracy or integrity of any part of the work are appropriately investigated and resolved.

## Conflict of Interest

The authors declare that the research was conducted in the absence of any commercial or financial relationships that could be construed as a potential conflict of interest.
