# Supplementary material for: Exploring Pediatric Tele-Rheumatology Practices During COVID-19: A Survey of the PRCOIN Network
Source: Front Pediatr. 2021 Mar 4;9:642460. doi: 10.3389/fped.2021.642460 (PMC7970043; doi:10.3389/fped.2021.642460)
Supplement: Supplementary file 1 [file Data_Sheet_1.DOCX]

Supplementary Material

**Survey**

**COVID-19 Experience at PR-COIN Sites**

The purpose of this survey is to understand the impact of COVID-19 on pediatric rheumatology clinic ambulatory visits (for all rheumatology patient subgroups). We would like to invite one respondent from your center to provide high level information reflecting your center's experience during COVID-19.

**GENERAL QUESTIONS**

**Which PR-COIN center do you represent?**

- Boston Children's Hospital
- Children's Hospital of Philadelphia
- Children's Mercy Kansas City
- Children's of Alabama
- Cincinnati Children's Hospital Medical Center
- Cohen Children's Medical Center of New York
- Hackensack University Medical Center
- Hospital for Special Surgery
- Levine Children's Hospital
- McMaster Children's Hospital
- Medical College of Wisconsin
- Medical University of South Carolina
- Nationwide Children's Hospital
- Nemours—Delaware
- Nemours—Orlando
- Penn State Hershey Children's
- Phoenix Children's Hospital
- Stanford Children's Health
- The Hospital for Sick Children
- University of Minnesota
- University of Mississippi

**What is the composition of your rheumatology team?**

- Attending pediatric rheumatologists
- NP
- MA
- RN
- Fellows
- PT
- OT
- Other

**How many attending pediatric rheumatologists practice at your site?**

**How many NPs practice at your site?**

**How many MAs practice at your site?**

**How many RNs practice at your site?**

**How many fellows are at your site?**

**How many PTs practice at your site?**

**How many OTs practice at your site?**

**Please list and quantify the number of other individuals who practice at your center? e.g. Position-#**

**TELEMEDICINE**

For the purposes of this survey telemedicine is defined as "remote diagnosis and treatment of patients by means of telecommunications technology". Telecommunications can be conducted by platforms including phone, live videoconferencing, or mobile applications.

**What telemedicine platforms has your site been using during COVID-19?**

- We do not perform telemedicine visits
- Telephone
- Videoconferencing system
- Telemedicine center (patient travels to a satellite center which is located closer to their home)
- Electronic health record patient portal
- Other

Please specify what other telemedicine platform your site uses.

**What video platforms does your site use?**

- MS Teams
- Zoom
- Jabber
- WhatsApp
- FaceTime
- Skype
- Doximity
- Other

Please specify what other video conferencing platforms your site uses

**What EHR patient portal does your site use?**

- MyChart (Epic)
- HealthELife (Cerner)
- Centricity Patient Portal (GE Centricity)
- FollowMyHealth (Allscripts)
- Other

Please specify what other EHR patient portal your site uses.

**Approximately what proportion of visits were conducted using telemedicine prior to COVID-19?**

- 0
- ≤10%
- more than 10 to ≤25%
- more than 25 to ≤50%
- more than 50% to ≤75%
- more than 75% to ≤100%

**Approximately what week did your clinic start using telemedicine because of COVID-19?**

**Approximately what proportion of visits were conducted using telemedicine during the initial stages (i.e. week of or immediately after closures) of COVID-19?**

- 0
- ≤10%
- more than 10 to ≤25%
- more than 25 to ≤50%
- more than 50% to ≤75%
- more than 75% to ≤100%
- 100%

(This question is divided into phases as different sites are currently different stages)

**Approximately what proportion of visits were conducted using telemedicine during (i.e. once telemedicine mechanisms were established) COVID-19?**

- 0
- ≤10%
- more than 10 to ≤25%
- more than 25 to ≤50%
- more than 50% to ≤75%
- more than 75% to ≤100%
- 100%

**Approximately what proportion of visits were conducted using telemedicine during phased reopening (i.e. this assumes your institution has started a phased re-opening) COVID-19?**

- 0
- ≤10%
- more than 10 to ≤25%
- more than 25 to ≤50%
- more than 50% to ≤75%
- more than 75% to ≤100%
- 100%
- Our institution has not started a phased reopening yet

**What types of patients are you seeing via telemedicine?**

- New patients
- Follow-up patients

**Please list the reasons that you would see a patient in person rather than see them over telemedicine.**

- Needs laboratory visit
- Active disease
- Joint injection
- Worsening condition
- New patient
- Parent request/desire
- Evidence of new rheumatic disease
- Anticipate hospitalization
- Other

What other circumstances would prompt you to see a patient in person vs. telemedicine?

**How has the volume of your patient encounters changed as a result of switching to telemedicine during COVID-19?**

- Decreased more than 50%
- Decreased more than 25% to ≤50%
- Decreased more than 10% to ≤25%
- About the same +/- 10%
- Increased more than 10% to ≤25%
- Increased more than 25% to ≤50%
- Increased more than 50%
- Not sure

**DOCUMENTATION**

**What information is documented (as close as to in person visit as possible) during your telemedicine visits?**

- Visit conducted using telemedicine
- Weight
- Height
- Joint count
- Physician Global Assessment
- Patient Global Assessment
- Patient Reported Outcomes
- Medication reconciliation
- Allergy review
- Medication refills
- Laboratory results
- Date of last eye examination
- Disease activity (e.g. JADAS, SLEDAI)
- Treatment target(s)
- Other

What other information do you collect during the telemedicine visit?

**Approximately what proportion of your providers consistently use PGALS to document musculoskeletal exams in patients with JIA?**

- 0
- up to ≤25%
- more than 25% to ≤50%
- more than 50% to ≤75%
- more than 75% to < 100%
- 100%

**What patient reported outcomes (PROs) do you collect for telemedicine visits?**

- We don't collect any PROs
- Patient global assessment
- CHAQ
- PROMIS
- PedsQL
- JAMAR
- Pain intensity
- Morning stiffness
- Patient self-reported joint count
- Other

Please list other PRO(s) that you collect.

**How does your site collect PROs?**

- Sent prior to telemedicine visit using EMR patient portal e.g. MyChart
- Sent prior to telemedicine visit by email
- Sent prior to telemedicine visit by mail
- Verbally collected during telemedicine appointment
- Other

What other method does your site use to collect PROs?

**On average, what is your PRO completion rate for telemedicine visits during COVID-19?**

- 0
- up to ≤25%
- More than 25% to ≤50%
- More than 50% to ≤75%
- More than 75% to < 100%
- 100%

**PATIENT MATERIALS**

**How are patients asked/what instructions are provided to patients to prepare for their telemedicine appointment?**

**What information does the patient receive at the end of their telemedicine visit?**

- Prescription
- After visit summary
- Referrals
- Other

What other materials do your patients receive after their telemedicine visit?

**How does your patient receive these materials?**

- App associated with patient's EMR
- Email
- Fax
- Mail
- Other

What other method do you use to provide patients information about their visit?

**RESEARCH**

**Does your center conduct research during telemedicine visits?**

- Yes
- No
- Currently in process of implementation

**How does your site integrate research into telemedicine visits? (i.e. identification, consent, follow-ups)**

**YOUR TEAM'S EXPERIENCE WITH TELEMEDICINE DURING COVID-19**

**What benefits has your team encountered during your telemedicine visits during COVID-19?**

- Less cancellations/no shows
- Decreased patient wait times (i.e. shorter time to schedule an appointment)
- Decreased clinic visit length (i.e. time spent with healthcare provider)
- No travel
- More convenient
- Continuity of care for families who are hesitant to come for in person visits
- Other

What other benefits has your team observed with telemedicine visits?

**What challenges has your team encountered during your telemedicine visits during COVID-19?**

- Assessing disease activity
- Limited ability to perform physical exams
- Safety labs being performed at recommended interval
- Providing multidisciplinary care
- Patient education
- Communicating after-visit instructions/making follow-up visits
- Access to technology
- Licensure
- Reimbursement
- Adequate internet bandwidth
- Other

What other challenges has your team encountered?

**Select the statement that your team agrees with the most:**

**As a healthcare provider, our team feels that telemedicine has met our needs.**

- Strongly disagree
- Disagree
- Neither agree nor disagree
- Agree
- Strongly agree

**Our team feels that telemedicine has met the needs of our patients.**

- Strongly disagree
- Disagree
- Neither agree nor disagree
- Agree
- Strongly agree

**FUTURE OF TELEMEDICINE**

**Do you believe that utilization of telemedicine in pediatric rheumatology should continue following the resolution of COVID-19 state of emergency?**

- Yes
- No

**In the future, assuming no licensure/reimbursement barrier, what percent of ESTABLISHED patient visit volume could be offered safely and effectively via telemedicine?**

- 0%
- 1-10%
- >10-25%
- >25-50%
- >50-75%
- >75-100%

**In the future, assuming no licensure/reimbursement barrier, what percent of NEW patient visit volume could be offered safely and effectively via telemedicine?**

- 0%
- 1-10%
- >10-25%
- >25-50%
- >50-75%
- >75-100%

**ADDITIONAL COMMENTS**

**Please share any other comments that you would like us to know about your COVID-19 telemedicine experience.**
